# Biodiversity of rodent anelloviruses in China

**DOI:** 10.1038/s41426-018-0037-x

**Published:** 2018-03-21

**Authors:** Jiang Du, Yuhui Li, Liang Lu, Dandan Zheng, Bo Liu, Li Yang, Haoxiang Su, Jie Dong, Lilian Sun, Yafang Zhu, Jian Yang, Fan Yang, Xiaobing Zhang, Qiyong Liu, Zhiqiang Wu, Qi Jin

**Affiliations:** 1 MOH Key Laboratory of Systems Biology of Pathogens, Institute of Pathogen Biology, Chinese Academy of Medical Sciences & Peking Union Medical College, Beijing, 100730 China; 2 State Key Laboratory for Infectious Diseases Prevention and Control, National Institute for Communicable Disease Control and Prevention Chinese Center for Disease Control and Prevention, Beijing, 102206 China; 3 Collaborative Innovation Center for Diagnosis and Treatment of Infectious Diseases, Hangzhou, 310058 China

Anelloviruses comprise a group of non-enveloped circular single-stranded DNA viruses with genomes of 2–4 kb. Anelloviruses were first reported in 1997 in a Japanese patient with post-transfusion hepatitis of unknown etiology^1^. Many human and animal anelloviruses have since been characterized, including strains in pigs, rodents, pine martens, non-human primates, sea lions, wild boar, cats, camels, and dogs^2–4^. The current taxonomic classification of *Anelloviridae* by the International Committee on Taxonomy of Viruses includes at least 12 genera and 65 species. Although the pathogenic potential of anelloviruses remains controversial^5^, some genotypes or genogroups are suggested to be pathogenic^6^ and some have been related to the progression of syndromes caused by other viral agents^7^.

Rodents are important natural hosts for the circulation of diverse viruses in the natural environment, including hantaviruses and arenaviruses. Rodent anelloviruses have previously been reported in the UK^4, 8^, but a worldwide survey of anelloviruses in various rodents remains lacking.

In this study, we performed a systematic survey of rodent-borne anelloviruses using virome data from rodents collected in 20 provinces throughout China from sites covered by the National HFRS (hemorrhagic fever with renal syndrome) Surveillance Network. The animals belonged to 42 species in the order Rodentia. Samples of the same species from the same sampling site were pooled together and processed using viral particle-protected nucleic acid purification, as described previously^9^. They were subjected to viral nucleic acid library construction, next-generation sequencing, metagenomic analysis, and taxonomic assignments. A total of 85,153 reads were classified into the family *Anelloviridae*. The anellovirus-positive pools in multiple rodent species from different provinces are presented in Fig. 1a. Eighteen species of Muridae and 12 species of Cricetidae carried anelloviruses, accounting for 84.48 and 75% of sample pools of each family, respectively. Three species of Sciuridae, three of Dipodidae, and one of Chinchillidae also carried anelloviruses. The genera *Rattus*, *Mus*, *Niviventer*, and *Apodemus* in the family Muridae contain numerous species of domestic rodents and had a higher anellovirus-positive rate at the pool level. Sample pools of some species from different provinces, including *Rattus flavipectus*, *Niviventer*, and *Apodemus peninsulae*, were always anellovirus-positive. We analyzed fewer samples of Sciuridae and Dipodidae, but the read data showed that they were also hosts of anelloviruses.

**Fig. 1:**
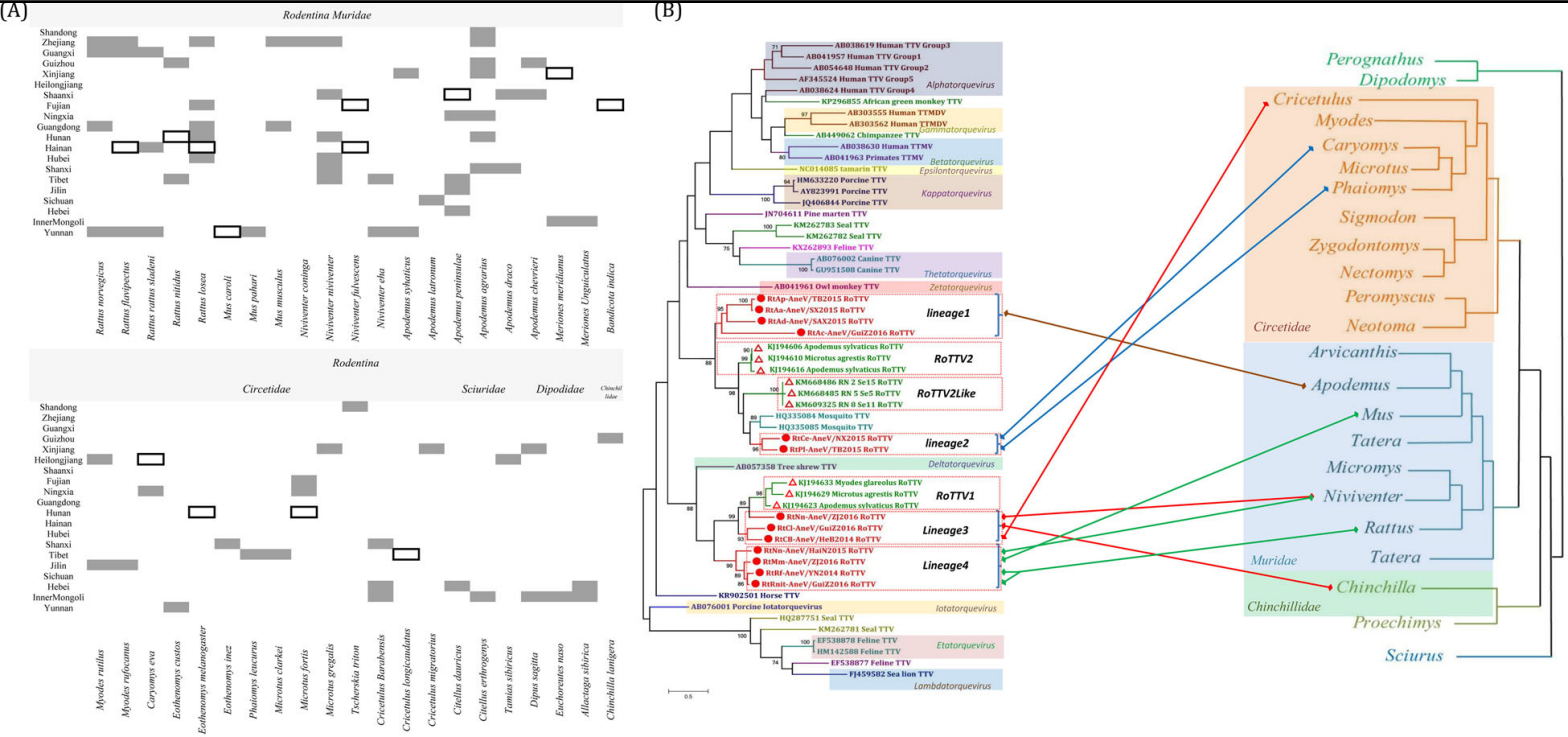
**a** Anellovirus-positive pool samples of rodents in China. Blank spaces represent absent samples. The black frames represent anellovirus negativity, and dark shading represents anellovirus positivity in each species and province. **b** Combined phylogenetic trees for anelloviruses and rodent hosts. The anellovirus phylogenetic tree based on the complete ORF1 protein sequences was constructed using MEGA6 with the mtREV + F + G + I model after alignment with the MUSCLE package, with 1000 bootstrap replicates. The rodent phylogenetic tree based on mitochondrial-cyt b was drawn from genus to family according to previous reports^12^. The viruses found in the current study are indicated by red circles (). RoTTV1, RoTTV2, and RoTTV-like are indicated by red triangles (). Text and box color demarcations represent different genera of Anelloviridae and different families of Rodentia, respectively. The colors of these lines represent different RoTTV clusters

Specific inverse nested primers were designed based on partial viral genomic sequences obtained by metagenomic analyses for polymerase chain reaction confirmation of each pooled sample. According to the amino acid identity, representative positive samples for each virus were selected for genome sequencing. Eleven representative complete genomes and two partial genomes were amplified. Thirteen new rodent torque teno virus (RoTTV) strains were sequenced from samples from the families Muridae, Cricetidae, and Chinchillidae collected from different regions of China (Supplementary Table S1) (MF684737 to MF684749).

Complete amino acid sequences of ORF1 were aligned for phylogenetic analyses. Phylogenetic analyses showed that the anelloviruses from rodents identified in this study clustered in four lineages (Fig. 1b). Lineage 1 contained four strains that showed amino acid sequence identity with other anellovirus genera for ORF1 of 6.2–17.7%. Lineage 1 strains clustered with RoTTV2(UK), RoTTV2-like, and mosquito TTV(USA). The amino acid identities of ORF1 between Lineage 1 strains and RoTTV2, RoTTV2-like, mosquito TTV, and Lineage 2 strains were 19.3–33.6%, 16.6–31.6%, 19.1–31.9%, and 19.8–30.9%, respectively. The amino acid identities of ORF1 among the four Lineage 1 strains ranged from 15.2–70.4%. The Lineage 2 strains were closely related to mosquito TTV, RoTTV2, and RoTTV2-like. The amino acid identities of ORF1 of Lineage 2 strains with mosquito TTV, RoTTV2, RoTTV2-like, and other genera were 39.2–40.8%, 41.9–43.2%, 34.8–36.5%, and 8.4–19.5%, respectively. The amino acid identity of ORF1 between the two Lineage 2 strains was 51.3% (Supplementary Table S2).

Lineage 1 formed a cluster, with all of their hosts belonging to the genus *Apodemus*, family Muridae (*Apodemus peninsulae*, *Apodemus agrarius*, *Apodemus draco*, and *Apodemus chevrieri*) (Fig. 1b), showing the close host relationship of Lineage 1 viruses. Lineage 2 viruses contained two strains isolated from *Caryomys eva* and *Phaiomys leucurus*, family Cricetidae (Fig. 1b). The host species and geographical locations of Lineage 1 and Lineage 2 strains differed from those of RoTTV2 and RoTTV2-like. Thus, Lineage 1 and Lineage 2 strains were suggested to be new genera in the family *Anelloviridae*, and evolutionary analysis suggested that they had a common ancestor.

Lineage 3 and Lineage 4 strains clustered with RoTTV1 (UK) and formed an independent cluster. ORF1 of Lineage 3 strains showed 33.8–55.2%, 21.2–29.8%, and 5.2–19.8% amino acid identities with RoTTV1, Lineage 4, and other genera, respectively. The amino acid identities of ORF1 among three Lineage 3 strains ranged from 35.6 to 52.1%. The amino acid identities of ORF1 of Lineage 4 strains with RoTTV1 and other genera were 23.3–30% and 6.2–19.8%, respectively, and the amino acid identities among three Lineage 4 strains ranged from 46.8–88% (Supplementary Table S2).

Lineage 3 contained three strains isolated from *N. niviventer* (Muridae), *Chinchilla lanigera* (Chinchillidae), and *Cricetulus barabensis* (Cricetidae). Lineage 4 contained four strains isolated from *N. niviventer* (Muridae), *Mus musculus* (Muridae), *R. flavipectus* (Muridae), and *Rattus nitidus* (Muridae) (Fig. 1b). Although the host species and geographical locations of Lineage 3 and Lineage 4 strains were different from those of rodent RoTTV1, the relatively high homology between Lineage 3, Lineage 4, and RoTTV1 suggested that viruses in this cluster were widespread in different locations with diverse hosts. Lineage 3 strains, together with RoTTV1 and Lineage 4 strains, were suggested to represent a new genus of *Anelloviridae*.

The pathogenicity of anelloviruses remains poorly understood because of the lack of a suitable model cell line or animal to support the viral cycle. Neither laboratory mice nor other laboratory animals, including primates, infected with human TTV have proven successful^10^; thus far, pigs have proved to be the best model species for studying anellovirus infections^11^. However, large experimental animals such as pigs are costly to treat and maintain in terms of reagents and resources. The results of the current study indicated that *M. musculus* carries the rodent anellovirus strain RtMm-AneV/ZJ2016 (Lineage 4), suggesting that laboratory mice of the same species could also be infected by this strain for use in studying the anellovirus viral cycle and pathogenicity. The financial and technical benefits of such a small animal model would facilitate further anellovirus-related research.

We outlined the rodent anellovirus viromes and identified 13 novel anellovirus strains in China. These data also provide a meaningful basis for the identification and tracking of potential rodent-origin anelloviruses.

## Electronic supplementary material

Supplementary Table S1(XLSX 9 kb)

Supplementary Table S2(XLSX 30 kb)
